# A compositional analysis of time spent in sleep, sedentary behaviour and physical activity with all-cause mortality risk

**DOI:** 10.1186/s12966-021-01092-0

**Published:** 2021-02-06

**Authors:** Anna E. Clarke, Ian Janssen

**Affiliations:** 1 Department of Public Health Sciences, Queen’s University, Kingston, ON K7L 3N6 Canada; 2 School of Kinesiology and Health Studies, Queen’s University, Kingston, ON K7L 3N6 Canada

**Keywords:** Compositional data analysis, Sleep, Sedentary behaviour, Physical activity, Public health, Survival analysis, 24-h movement behaviours

## Abstract

**Background:**

Daily time spent in sleep, sedentary behaviour (SED), light intensity physical activity (LIPA), and moderate-to-vigorous intensity physical activity (MVPA) are compositional, co-dependent variables. The objectives of this study were to use compositional data analysis to: (1) examine the relationship between the movement behaviour composition (daily time spent in sleep, SED, LIPA and MVPA) and all-cause mortality risk, and (2) estimate the extent to which changing time spent in any given movement behaviour (sleep, SED, LIPA, or MVPA) within the movement behaviour composition was associated with changes in risk of all-cause mortality.

**Methods:**

2838 adult participants from the 2005–2006 cycle of the U.S. National Health and Nutrition Examination Survey were studied using a prospective cohort design. Daily time spent in SED, LIPA and MVPA were determined by accelerometer. Nightly time spent sleeping was self-reported. Survey data were linked with mortality data through to the end of December 2015. Compositional data analysis was used to investigate relationships between the movement behaviour composition and mortality.

**Results:**

The movement behaviour composition was significantly associated with mortality risk. Time spent in MVPA relative to other movement behaviours was negatively associated with mortality risk (HR = .74; 95% CI [.67, .83]) while relative time spent in SED was positively associated with mortality risk (HR = 1.75; 95% CI [1.10, 2.79]). Time displacement estimates revealed that the greatest estimated changes in mortality risk occurred when time spent in MVPA was decreased and replaced with sleep, SED, LIPA or a combination of these behaviours (HRs of 1.76 to 1.80 for 15 min/day displacements).

**Conclusions:**

The daily movement behaviour composition was related to mortality. Replacing time in MVPA or SED with equivalent time from any other movement behaviour was associated with an increase and decrease in mortality risk, respectively.

**Supplementary Information:**

The online version contains supplementary material available at 10.1186/s12966-021-01092-0.

## Background

Sleep, sedentary behaviour (SED), light intensity physical activity (LIPA), and moderate-to-vigorous intensity physical activity (MVPA) are movement behaviours and time spent in these behaviours adds up to a constant 24-h per day sum [1, 2]. Short and long sleep durations and greater time spent in SED have been associated with an increased risk of mortality whereas more time spent being physically active has been associated with reduced mortality risk [3–5]. Previous studies have largely explored associations between individual movement behaviours and mortality in isolation. Recently, researchers have explored how multiple movement behaviours, such as the combination of high SED and low MVPA, influence mortality [6, 7].

The relationships between multiple movement behaviours with morbidity and mortality have generally been studied using standard regression techniques that include movement behaviours as *independent* variables in a regression model [6, 7]. While this has shed additional light on how combinations of movement behaviours influence health, as explained elsewhere [1, 2, 8, 9], there are numerous limitations to the statistical approaches used in these studies and using standard regression techniques to study movement behaviours is problematic and may lead to erroneous conclusions [1, 2, 8, 9]. To avoid these problems, compositional data analysis (CoDA) can be used. CoDA is a statistical approach suitable for co-dependent variables that are relative components of a finite sum [10], such as time spent in movement behaviours adding up to the 24-h day [1, 2, 8, 9]. As noted in a new systematic review, the few 24-h movement behaviour studies that have used CoDA have largely been cross-sectional in nature and limited to examining a small selection of health indicators such as adiposity, cardiometabolic risk factors, and measures of mental health [11]. A key conclusion from this review is that the daily movement behaviour composition is associated with a variety of health outcomes. The authors also reported that relative time spent in MVPA was favourably associated with health outcomes and associations for time reallocation estimates were consistently favourable when time was reallocated to MVPA (from other movement behaviours) and unfavourable when MVPA was removed and replaced with sleep, SED or LIPA. These estimates also revealed favourable associations for health outcomes when time was removed from SED and replaced with LIPA or MVPA. Subsequent to the conduct of the aforementioned systematic review [11], several new cross-sectional studies have examined the association between the 24-h movement behaviour composition and health indicators [12–20]. However, to our knowledge, only one longitudinal study has used CoDA to examine the association between the full 24-h movement behaviour composition (i.e., time spent in sleep + SED + LIPA + MVPA) and health [21]. The authors found that the composition of time spent in sleep, SED, LIPA and MVPA was significantly associated with risk of all-cause mortality during a 5–6 year follow-up. They showed in graphic format that replacing time spent in any movement behaviour with MVPA was associated with a lower mortality risk. Replacing time spent in sleep or SED with LIPA and replacing time spent in SED with sleep were also favourable. A key limitation of that study was the inclusion of participants with as little as one valid day of accelerometry data. A single day may not accurately or reliably reflect participants’ habitual movement patterns and could result in misclassification of their movement behaviour composition [22, 23]. Studies have suggested that 4 days of accelerometer data are necessary to obtain reliable measures of physical activity (ICC ≥ 0.80) [22, 23]. Another limitation was the short follow-up and number of deaths, as such the study may have been subject to increased reverse causality bias [24] and may not have had adequate power to detect associations for all movement behaviours. Furthermore, the time displacement findings were only presented graphically, so readers are unable to extract specific hazard ratio values corresponding to time displacement estimates. Confounder selection in this study is also concerning. A comprehensive measure of the diet was missing, yet two health status covariates that lie on the causal pathway between movement behaviours and mortality were included.

The objectives of this study were to use a CoDA approach to: (1) investigate whether the composition of time spent in sleep, SED, LIPA and MVPA was associated with all-cause mortality risk; and, (2) estimate the degree to which changing time in any given movement behaviour (sleep, SED, LIPA, or MVPA) within the movement behaviour composition was associated with changes in risk of all-cause mortality.

## Methods

### Data source and study participants

This study used data from the 2005–2006 cycle of the U.S. National Health and Nutrition Examination Survey (NHANES). The NHANES is a series of surveys and physical health measures that aim to assess the health and nutritional status of the American population [25, 26]. The NHANES surveys a nationally representative sample using a stratified, multistage probability design of civilian, non-institutionalized participants. NHANES data are publicly accessible and include survey and interview data collected in a home interview, physical and biological measures obtained in a mobile exam centre visit and follow-up record linkage for mortality. Unlike other cycles of the NHANES, the 2005–2006 cycle collected objective waking movement behaviour data and information on sleep duration, which allowed us to evaluate the entire 24-h movement behaviour composition. Ethics approval was obtained from the National Center for Health Statistics. Participants provided written, informed consent.

To be included in this study, NHANES participants were required to be at least 20 years old and not pregnant at the time of their mobile examination centre visit and provided consent for mortality follow-up (*n* = 4641). Participants were excluded if they had less than 4 days of valid accelerometry data (defined as ≥10 h of wear time, *n* = 1691) or were missing self-reported sleep data (*n* = 4). One participant missing education covariate data was excluded. Participants who suffered an accidental death (*n* = 15) during the follow-up period were excluded because these were not expected to be related to the movement behaviour composition. Furthermore, participants who died prior to 1 year of follow up (*n* = 27) were excluded to maintain a relevant exposure time window and reduce the potential for misclassification of habitual movement behaviour patterns which may have been altered close to death. Finally, 65 participants who were long sleepers (i.e., reported sleeping 10 or more hours per night [27]) were excluded since there were not enough long sleepers to power a separate analysis. This left a final study sample of 2838 adult participants.

### Exposure: movement behaviour composition

The exposure of interest was participants’ baseline movement behaviour composition as assessed in 2005–2006. Participants were asked to wear a uniaxial accelerometer (Actigraph PAM-7164, Pensacola, FL) on their right hip for 7 consecutive days [28]. Participants were instructed to remove the accelerometers at bedtime and to keep them dry (e.g., remove when swimming or bathing). The accelerometers recorded movement intensity (magnitude of acceleration) over 10,080 consecutive 1-min intervals – one interval for each minute of the week - and these data were used to determine time spent in SED, LIPA, and MVPA. Non-wear time (i.e., when participants were not wearing the accelerometer) was defined as ≥90 consecutive minutes where the intensity count was equal to zero, allowing for up to 2 consecutive minutes with intensity counts between 1 and 99 [29]. Invalid accelerometer collection days (i.e., wear time < 10 h) and participants with insufficient valid accelerometer data (i.e., < 4 valid days) were removed [29, 30]. The remaining accelerometer count data was used to determine average daily time spent in SED (< 100 counts per minute), LIPA (100–2019 counts per minute), and MVPA (≥2020 counts per minute) [29, 31].

Information on sleep duration to the nearest hour was gathered from responses to the computer-assisted personal interview item “How much sleep do you actually get at night on weekdays or workdays?”. Responses were rounded to the nearest hour and could range from 1 to 12 h/night. Average sleep duration was expressed as a proportion of 24 h. The remaining proportion of 24 h (i.e. 24 h minus self-reported sleep duration), was used to normalize average time spent in SED, LIPA and MVPA per day, which was then expressed as a proportion of the full 24-h day. There were 8 zero values for average daily time spent in MVPA and a single zero value for SED. These zero values were assigned the smallest possible value that we could have detected (i.e., 1 minute per week).

### Outcome: mortality

NHANES survey data were linked with mortality data through December 31st, 2015 using probabilistic record matching with the National Death Index, a database of all U.S. deaths [32]. The National Death Index has a sensitivity of 87–98% and specificity of 99–100% [32, 33]. Other sources of mortality information were also used including linkage with Social Security administration and active follow-up. The public-use linked mortality file was used to obtain vital status (assumed alive or assumed deceased), leading underlying cause of death and follow-up time in person months from the date of the mobile examination centre visit to the date of death or end of the follow-up period.

### Covariates

Covariates were investigated as possible confounders based on their known association with mortality and at least one movement behaviour, but only if they did not lie on the causal pathway. Covariate measures were obtained using self-reported questionnaires, in person interviews (including 24-h food recalls), and direct physical measures. Several potential covariates were considered including age (continuous), sex (male or female), race/ethnicity (non-Hispanic white, non-Hispanic black, Mexican-American, other Hispanic, other race including multi-racial), smoking status (non-smoker, former smoker, current smoker), highest level of education (<high school, high school, college degree), family poverty-to-income ratio, body mass index (BMI) category, alcohol consumption, and diet quality. Family poverty-to-income ratio was determined by dividing family income by poverty guidelines specific to family size, year and state. Poverty-to-income-ratio values were categorized into weighted quartiles. BMI was calculated from height and weight measurements collected by health technicians and categorized into underweight (< 18.5 kg/m^2^), normal weight (18.5–24.9 kg/m^2^), overweight (25–29.9 kg/m^2^), obese class I (30–34.9 kg/m^2^), and obese class II or higher (≥35 kg/m^2^) [34]. Alcohol consumption was categorized as non-drinkers, light-to-moderate drinkers (1–14 drinks/week for men, 1–7 drinks/week for women), and heavy drinkers (> 14 drinks/week for men, > 7 drinks/week for women) based on existing guidelines [35]. A healthy eating index score, a diet quality tool that measures conformance with the 2010 Dietary Guidelines for Americans, was derived for each participant using several dietary variables that were captured from two 24-h food recall assessments and a food frequency questionnaire [36]. Weighted quartiles of the health eating index scores were calculated. A ‘missing’ category for participants with missing values was created for some variables (family poverty-to-income ratio [*n* = 116], BMI [*n* = 11], alcohol consumption [*n* = 179] and diet quality [*n* = 91]) to avoid removing these observations from the analyses and help preserve statistical power.

### Analysis strategy

Statistical analyses were performed in SAS version 9.4 (SAS Inc., Carry, NC). NHANES survey design and sample weights were accounted for in the analyses using SURVEY functions. Conventional descriptive statistics were derived (e.g., means, proportions, death rates) for the variables of interest. CoDA was used to describe the movement behaviour variables, to determine their codependence, and to assess the association between the movement behaviour composition and its components with all-cause mortality risk. CoDA is suitable for data that make up portions of a finite whole (i.e., movement behaviours in a 24-h day) [8]. Geometric means for time spent in each movement behaviour were calculated (adjusted to collectively add to 100% or 24 h) as they better capture the central tendency of compositional data than conventional arithmetic means [1, 8].

The codependence between movement behaviours was assessed using pair-wise log ratio variances between all behaviours [e.g. variance of ln (sleep/SED)] and scaled to aid in interpretation [(${\mathcal{e}}^{-\frac{t^2}{2}}\Big)$ where t is any log ratio variance]. Values for these pair-wise log ratio variances could range from zero to one; values closer to one indicated a higher codependence. Cox proportional hazards regression models were used to examine the association between the movement behaviour composition and mortality risk. Prior to fitting the regression models, the movement behaviour variables were transformed from their natural space, the constrained simplex (i.e., a 24-h day) onto standard real space. For this step, *isometric log ratio (ilr)* transformations were used to express the movement behaviour composition as ratios of its parts (i.e., *absolute* time spent in sleep, SED, LIPA and MVPA). This transformation generated *ilr* coordinates in real space based on a sequential partition of one movement behaviour to the remaining movement behaviours (e.g., time spent in sleep *relative* to SED, LIPA and MVPA) [8]. Daily activity composition allotted into four parts (sleep, SED, LIPA and MVPA) was expressed as three *ilr* coordinates [*z*_*i*1_, *z*_*i*2_, *z*_*i*3_]. These *ilr* coordinates captured the combined distribution of all parts of the composition (time spent in sleep, SED, LIPA, and MVPA). For example, *ilr* coordinates for sleep’s relative contribution were found as follows:

$$ {Z}_{i1}=\sqrt{\frac{3}{4}}1\mathrm{n}\left(\frac{slee{p}_i}{\sqrt[3]{S{B}_i\times { MVP A}_i\times {LIPA}_i}}\right),{Z}_{i2}=\sqrt{\frac{2}{3}}1\mathrm{n}\left(\frac{S{B}_i}{\sqrt{MVP{A}_i\times {LIPA}_i}}\right), and\kern0.5em {Z}_{i3}=\sqrt{\frac{1}{2}}1\mathrm{n}\left(\frac{MVP{A}_i}{\sqrt{LIP{A}_i}}\right) $$

After movement behaviour data were *ilr* transformed, Cox proportional hazards regression models estimating survival were built using the corresponding set of three *ilr* coordinates for each movement behaviour and the confounding variables as explanatory variables. Overall maximum likelihood test statistics from the robust regression models were used to assess the significance of the entire movement behaviour composition. The coefficient and *p*-value corresponding to the first *ilr* coordinate variable were used to assess if that specific movement behaviour was significantly associated with mortality, *relative* to time spent in the remaining movement behaviours. Only the first *ilr* coordinate variable in each model was interpreted as it contained all the relevant information regarding a participant’s movement behaviour composition (e.g. sleep relative to the remaining movement behaviours) [1, 8]. The second and third *ilr* coordinate variables were used to fit the model, but not meaningfully interpreted. A backward elimination approach with a liberal *p*-value (0.20) was used to remove covariates unrelated to mortality. Statistical interactions by sex and age were investigated based on their a priori consideration as potential effect modifiers. This was done by including product terms between sex and age with each movement behaviour. The proportional hazards assumption held for the final models.

The Cox proportional hazard regression parameters of the *ilr* coordinates are difficult to interpret without a back transformation. To present the results of the Cox models in a more meaningful and interpretable way, we used the results from these models to estimate the extent to which displacing time spent in one movement behaviour with one or more of the remaining movement behaviours predicted changes in mortality [21]. For example, we estimated the hazard ratio (HR) associated with removing 15 min/day from the average time spent in MVPA and adding 15 min/day to the average time spent in sleep. Because compositional data are relative, the time displacement predictions must be made in relation to a reference point [21]. We used the study sample mean movement behaviour composition as the reference point.

## Results

### Descriptive characteristics

Descriptive baseline sociodemographic and health behaviour characteristics are summarized in Table 1. On average, participants were aged 46.4 years at baseline. There was a roughly even distribution of males and females (51.8% female). The majority (71.7%) were non-Hispanic white, high school graduates (85%), had overweight or obesity (66%), were non- or former smokers (79%), and were non-drinkers or light-to-moderate drinkers (86.6%).

**Table 1:** Participant characteristics

Variable	n	%	Weighted N	Weighted %
Sex				
Male	1461	51.5	98,445,733	48.2
Female	1377	48.5	105,912,178	51.8
Age				
20–39	829	29.2	77,648,076	38.0
40–60	995	35.1	81,118,121	39.7
≥ 60	1014	35.7	45,591,714	22.3
Race/Ethnicity				
Mexican American	573	20.2	16,113,144	7.9
Other Hispanic	82	2.9	7,252,096	3.5
Non-Hispanic white	1453	51.2	146,521,928	71.7
Non-Hispanic black	620	21.9	23,438,894	11.5
Other (including multi-racial)	110	3.9	11,031,849	5.4
Education				
< High school	726	25.6	30,712,002	15.0
High school	1513	53.3	118,267,386	57.9
College graduate	599	21.1	55,378,522	27.1
Poverty-to-income ratio				
Below poverty threshold (< 1)	395	13.9	17,970,937	8.8
At or above poverty threshold (≥1)	2327	82.0	180,200,584	88.2
Missing	116	4.1	6,186,389	3.0
BMI Category				
Underweight	36	1.3	3,117,177	1.5
Normal weight	817	28.8	65,554,605	32.1
Overweight	993	35.0	66,951,444	32.8
Obese class I	580	20.4	38,713,305	18.9
Obese class II +	401	14.1	29,269,908	14.3
Missing	11	0.4	751,472	0.4
Alcohol consumption				
Non-drinker	935	33.0	56,036,438	27.4
Light-to-moderate drinker	1536	54.1	120,840,686	59.1
Heavy drinker	188	6.6	16,811,619	8.2
Missing	179	6.3	10,669,167	5.2
Smoking status				
Non-smoker	1496	52.7	108,550,690	53.1
Former smoker	787	27.7	529,45,457	25.9
Current smoker	555	19.6	428,61,763	21.0
Healthy eating index				
Poor (< 50)	1250	44.1	95,280,809	46.6
Needs improvement (50–80)	1418	50.0	97,650,947	47.8
Good (80–100)	79	2.8	5,915,565	2.9
Missing	91	3.2	5,510,589	2.7

*Note*: n refers to the sample size while N refers to the number of participants that the sample represents

The geometric means for sleep, SED, LIPA and MVPA were (in hours:minutes per day) 6:58, 10:05, 6:40, and 0:17, respectively. The corresponding arithmetic means were 6:58, 8:33, 5:45, and 0:23. On average 29% of the 24-h day was spent sleeping, 42% was spent in SED, 28% was spent in LIPA, and 1% was spent in MVPA. Values for the pair-wise log ratio variances could range from 0 (lowest codependence) to 1 (highest codependence). The greatest pair-wise log ratio variances were between sleep and SED (0.99), followed by sleep and LIPA (0.98), and LIPA and SED (0.95). The lowest variances were between MVPA and SED (0.08), MVPA and sleep (0.17), and MVPA and LIPA (0.34). Therefore, MVPA had the least co-dependency with the other movement behaviours.

### Mortality risk

The follow-up length ranged from 1 to 11 years and was 9.7 years on average. During the 26,730 person-years of follow up, a total of 393 deaths occurred for a death rate of 147 per 10,000 person-years. Of the 393 deaths, 231 were in men and 162 were in women.

All-cause mortality risk estimates associated with each movement behaviour relative to the remaining movement behaviours are presented in Table 2. After backwards elimination, the final regression models were adjusted for age, sex, education, family poverty-to-income ratio, BMI, smoking, alcohol, and diet quality. The 24-h movement behaviour composition was significantly associated with mortality risk (*p* < .0001 for the overall model fit). The HRs for these models can be interpreted as proportional change in mortality risk associated with an increase in time spent in that behaviour relative to time spent in the remaining movement behaviours. Time spent in MVPA relative to the other movement behaviours was associated with a reduction in mortality risk (HR = .74; 95% CI = .67, .83, *p* < 0.0001). Relative time spent in LIPA was not associated with a significant reduction in mortality risk (HR = .75; 95% CI = .54, 1.04, *p* = .08). Relative time spent in SED was associated with an increase in mortality risk (HR = 1.75; 95% CI = 1.10, 2.79, *p* = 0.02). Relative time spent in sleep was not associated with mortality risk (HR = 1.02; 95% CI = .64, 1.63, *p* = 0.90).

**Table 2:** Compositional Cox regression model estimates

Model *p*-value	Sleep		SED		LIPA		MVPA	
	HR (95% CI)	*p*-value	HR (95% CI)	*p*-value	HR (95% CI)	*p*-value	HR (95% CI)	*p*-value
<.0001	1.02 (.64, 1.63)	0.9	1.75 (1.10, 2.79)	0.02	0.75 (.54, 1.04)	0.08	0.74 (.67, .83)	<.0001

Note: Adjusted for age, sex, education, family poverty-to-income ratio, BMI, smoking status, alcohol consumption, and diet quality. Hazard ratios can be interpreted as proportional change in mortality risk associated with an increase in time spent in that behaviour relative to time spent in the remaining movement behaviours

### Sensitivity analysis

For sex, none of the interaction terms were statistically significant. For age, only the interaction term containing LIPA relative to the remaining movement behaviours was statistically significant (*p* = .002). Based on existing literature, we hypothesised that this difference in the strength of the association between relative LIPA and mortality across different ages was attributable to variations in MVPA levels and not truly a differential association [37]. Indeed, in the present study when adults accumulated similar amounts of MVPA per day, associations for LIPA relative to the remaining movement behaviours were in the same direction regardless of age. For example, when participants obtained < 5 min per day of MVPA, relative LIPA was associated with lower mortality risk among both participants under 60 years old and participants 60 years of age and older. In contrast, when participants obtained 15 or more minutes per day of MVPA on average, this was not observed. As such, analyses are presented in all ages combined. Finally, we re-ran the compositional Cox models after included all 3384 participants with ≥1 valid day of accelerometer data. The HR were similar to those observed in the analysis based on the 2838 participants with ≥4 valid days of accelerometer data (Table 2). Specifically, the HR were 1.11 (95% CI = .73, 1.69, *p* = .59) for relative time spent in sleep, 1.86 (95% CI = 1.21, 2.88, *p* = .01) for relative time spent in SED, .65 (95% CI = .46, 0.90, *p* = .01) for relative time spent in LIPA, and .75 (95% CI = .67, .84, *p* < .0001) for relative time spent in MVPA.

### Compositional isometric substitution modelling

**Fig. 1:**
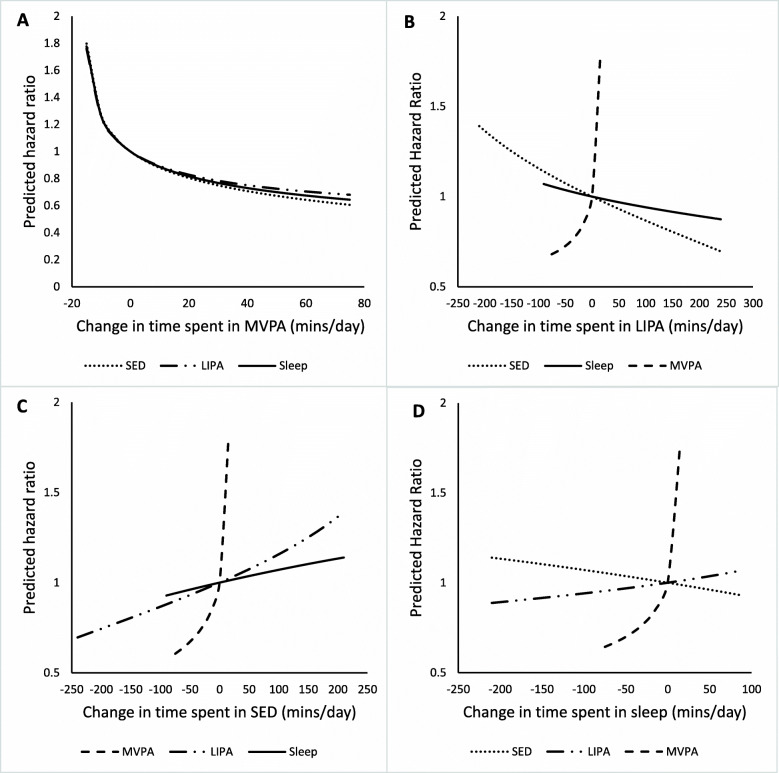
Estimated HRs associated with hypothetical time displacements from one movement behaviour to another. All estimates have been adjusted for age, gender, education, family poverty-to-income ratio, BMI, smoking status, alcohol consumption and diet quality. HRs reflect the hypothetical change in estimated mortality risk associated with reallocating time spent in a) MVPA, b) LIPA, c) SED, and, d) sleep based on parameter estimates from compositional regression. The difference in minutes/day are modelled around the mean movement behaviour composition (reference). Time is substituted between the movement behaviour on the x-axis and the movement behaviour indicated by the line. For example, Panel B shows estimated HRs associated with hypothetically changing the mean amount of time spent in LIPA. As more minutes are added to LIPA, it is estimated that the associated HR will increase if this time is taken from MVPA, but decrease if this time is taken from sleep or SED. Substitutions were not made beyond the range of the 2.5th to 97.5th percentile of minutes per day spent in each movement behaviour (e.g., no more than 75.4 min per day were added to the mean 16.8 min per day spent in MVPA)

**Fig. 2:**
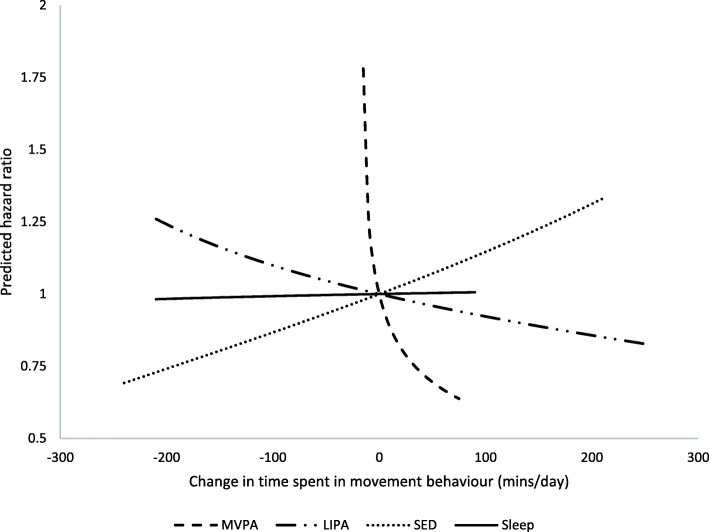
Estimated HRs associated with hypothetical time displacements between one and the remaining movement behaviours. All estimates have been adjusted for age, gender, education, family poverty-to-income ratio, BMI, smoking status, alcohol consumption and diet quality. HRs reflect the hypothetical change in estimated mortality risk associated with reallocating time between one movement behaviour and the remaining movement behaviours based on parameter estimates from compositional regression. The difference in min/day are modelled around the mean movement behaviour composition (reference). For example, if more time is hypothetically allocated to LIPA and removed from the remaining movement behaviours, the estimated hazard ratio for this association decreases. Substitutions were not made beyond the range of the 2.5th to 97.5th percentile of minutes per day spent in each movement behaviour (e.g., no more than 75.4 min per day were added to the mean 16.8 min per day spent in MVPA)

Figures 1 and 2 depict how parameter estimates from the regression models were used to estimate changes in mortality risk associated with equivalent time displacements from the mean movement behaviour composition. Figure 1 shows predicted changes in mortality risk associated with replacing time spent in one movement behaviour with another movement behaviour (e.g., removing 15 min/day of sleep and adding 15 min/day of SED). Figure 2 depicts estimated changes in mortality risk associated with displacing time spent in one movement behaviour to or from a combination of the remaining movement behaviours based on the relative proportional daily contribution of behaviours. For example, when 15 min/day was removed from the mean time spent in MVPA (Table 3, Fig. 2), 15 min/day was proportionally redistributed to the remaining movement behaviours such that 4.4 min were added to sleep, 6.4 min were added to SED and 4.2 min were added to LIPA. Values for estimated HRs associated with 15-min/day time displacements can be seen in Tables 3 and 4. Additional hazard ratios for time displacement estimates ranging from 5 min per day to 120 min per day can be found in Additional File 1.

**Table 3:** Estimated HRs for displacing 15 min between movement behaviours

Remove 15 min per day from	Add 15 min per day to			
	Sleep	SED	LIPA	MVPA
Sleep	–	1.01 (0.99, 1.03)	0.99 (0.99–0.99)	0.85 (0.81, 0.89)
SED	0.99 (0.97, 1.01)	–	0.98 (0.96, 1.00)	0.84 (0.78, 0.90)
LIPA	1.01 (1.01, 1.01)	1.02 (1.00, 1.04)	–	0.86 (0.82, 0.90)
MVPA	1.78 (1.47, 2.15)	1.80 (1.46, 2.22)	1.76 (1.46, 2.13)	–

Note: Data presented as HR (95% confidence interval). All estimates have been adjusted for age, gender, education, family poverty-to-income ratio, BMI, smoking status, alcohol consumption, and diet quality. Hazard ratios reflect the estimated change in mortality risk associated with reallocating 15 min from the movement behaviour in the column to the movement behaviour in the row using the mean movement behaviour composition as the reference

**Table 4:** Estimated HRs for displacing 15 min between one and the remaining movement behaviours proportionally

Movement behaviour	Remove 15 min of column behaviour and add to remaining behaviours	Add 15 min to column behaviour from remaining behaviours
Sleep	1.00 (0.99, 1.01)	1.00 (0.99, 1.01)
SED	0.98 (0.96, 1.00)	1.02 (1.00, 1.04)
LIPA	1.01 (1.00, 1.03)	0.99 (0.98, 1.00)
MVPA	1.78 (1.46, 2.17)	0.85 (0.80, 0.90)

Note: Data presented as HR (95% confidence interval). All estimates have been adjusted for age, gender, education, family poverty-to-income ratio, BMI, smoking status, alcohol consumption and diet quality. HRs reflect the estimated change in mortality risk associated with reallocating time from one movement behaviour to the remaining movement behaviours proportionally (or vice versa) using the mean movement behaviour composition as the reference

Estimates revealed an increased mortality risk associated with reallocating 15 min/day of MVPA into sleep (HR = 1.78; 95% CI = 1.47, 2.15), SED (HR = 1.80; 95% CI = 1.46, 2.22), LIPA (HR = 1.76; 95% CI = 1.46, 2.13), or a combination of sleep, SED, and LIPA (HR = 1.78; 95% CI = 1.47, 2.15). Conversely, reallocating 15 min/day more time into MVPA by taking 15 min/day out of sleep (HR = .85; 95% CI = .81, 0.89), SED (HR = .84; 95% CI = .78, .90), LIPA (HR = .86; 95% CI = .82, .90), or their combinations (HR = .85; 95% CI = .80, .90) was associated with a reduction in mortality risk. SED predictions showed that decreasing SED by 15 min/day was associated with a lower mortality risk when that time was reallocated into LIPA (HR = .98; 95% CI = .96, 1.00) or MVPA (HR = .84; 95% CI = .78, .90). Conversely, increasing SED by 15 min/day was associated with an increase mortality risk if that time was taken from MVPA (HR = 1.78; 95% CI = 1.47, 2.15). Reallocating 15 min per day to LIPA from sleep or SED was estimated to be associated with a lower mortality risk (HR = .99; 95% CI = .99, .99 and HR = .98; 95% CI:= .96, 1.00, respectively). The predictions also suggested that redistributing 15 min to sleep from SED was not associated with a change in mortality risk (HR = .99; 95% CI = 0.97, 1.01), but that when sleep replaced MVPA or LIPA it was associated with an increased mortality risk (HR = 1.78; 95% CI = 1.47, 2.15 and HR = 1.01; 95% CI = 1.01, 1.01). The estimates in Figs. 1 and 2 also suggest the relationship between changes in MVPA and mortality risk is asymmetrical. For instance, reallocating 15 min of MVPA to the remaining movement behaviours (Fig. 2) was associated with a larger increase in risk (HR = 1.78; 95% CI = 1.46, 2.170) than the reduction in risk (HR = .85; 95% CI = .80, .90) associated with redistributing 15 min of time from the remaining movement behaviours into MVPA.

## Discussion

This study used a CoDA framework to investigate the relationship between the daily movement behaviour composition and all-cause mortality. The composition of daily time spent in sleep, SED, LIPA and MVPA was significantly associated with mortality risk. The amount of time spent in MVPA relative to the remaining behaviours was significantly associated with a lower mortality risk whereas relative time spent in SED was significantly associated with an increased mortality risk. Estimates suggest reallocating time into MVPA from any of the other movement behaviours would be associated with a lower risk of mortality. Correspondingly, removing time from MVPA and adding this to any other movement behaviour would be associated with a greater risk of mortality. This relationship was asymmetrical, such that removing MVPA from the geometric mean MVPA value was associated with a greater reduction in mortality risk than the corresponding increase in mortality risk associated with adding MVPA to the geometric mean. This asymmetrical relationship is consistent with other studies that have examined relationships between MVPA relative to the remaining movement behaviours and health outcomes [1, 38]. There is a general consensus in the physical activity literature that small differences in MVPA are associated with large reductions in mortality risk at the lower end of the MVPA scale and that much larger differences in MVPA are needed at the higher end of the MVPA scale to further reduce mortality risk [39].

In 2019, McGregor et al. published the only study to date using CoDA to examine the relationship between the 24-h movement behaviour composition and all-cause mortality [21]. Similar to our paper, McGregor et al. found that the movement behaviour composition was significantly associated with mortality over 5 to 6 years in a sample of 1592 Americans 50–79 years of age. Though there was no statistically significant association between the amount of time spent in MVPA relative to the remaining behaviours and all-cause mortality in the fully adjusted model, the association did show a trend towards significance (*p* = .09) in a direction consistent with the present study. This difference can potentially be explained by the smaller sample size and shorter follow-up length in McGregor et al.’s study and over adjustment for two covariates (self-assessed health and physical limitations on movement) that lie on the causal pathway between movement behaviours and all-cause mortality. Typically, this kind of over adjustment can bias results towards the null, so true relationships may be obscured [40]. This paper further reports on associations between relative time spent in sleep, SED and LIPA and all-cause mortality, which are not reported by McGregor et al.

One other study by von Rosen et al. investigated the relationship between the waking movement behaviour composition and all-cause mortality but did not include sleep data [41]. Lacking information on sleep limited the ability of these authors to consider all co-dependent parts of the 24-h movement behaviour composition within a CoDA paradigm. They found that time spent in SED relative to LIPA and MVPA was significantly associated with greater mortality risk (HR = 2.24; CI = 1.41, 3.56). Replacing SED with LIPA or MVPA was associated with reductions in mortality risk. The present study expanded on this by measuring all parts of the full 24-h movement behaviour composition, including sleep.

Key implications of our study include recommendations to preserve time spent in MVPA over time spent in other movement behaviours, especially for those with low levels of MVPA. Among those spending little to no time in MVPA, large reductions in mortality risk can occur by increasing time in MVPA regardless of which other movement behaviour that time is taken from. Limiting time spent in SED when possible is also recommended; the estimates from the time displacements suggest that reducing SED would reduce mortality risk irrespective of what movement behaviour that time is reallocated to. Insufficient levels of sleep should be addressed by reallocating time to sleep from SED, but not from physical activity (especially MVPA).

Key strengths of this study include the use of device-based measures of physical activity and SED, its ability to examine the temporal relationship between the daily movement behaviour composition and all-cause mortality due to the prospective cohort study design, and the use of a novel analytic approach to analyze 24-h movement behaviour data in a large sample of adults. Moreover, this study used data from a nationally representative sample of Americans and the results should be generalizable to similar populations. There are also several limitations. Accelerometers, while an improvement over self-reported data, are imperfect measurement tools. MVPA stemming from participation in activities that are not step-based such as cycling, resistance training or swimming would have been underestimated. Due to the accelerometer cut-points, some LIPA, such as time spent standing, may have been misclassified as SED. Furthermore, accelerometers were removed at night and sleep duration was self-reported. As a result of this potential measurement error, non-differential misclassification could have occurred, which would likely have attenuated associations for the relative importance of these movement behaviours. Additionally, a large proportion of NHANES participants (36.4%) were removed from the analyses due to insufficient accelerometer data. If the relationship between daily movement behaviour composition and all-cause mortality risk was different between those with and without adequate accelerometer data, our results may be biased.

More research is needed applying CoDA techniques in movement behaviour research to further explore associations with health. For example, exploring potential differences in work-related SED and leisure-time and/or screen-time related SED. This could help elucidate possible differential effects of separate domains of SED. LIPA could also be broken up into lighter intensity LIPA and higher intensity LIPA to delve further into the importance of various behaviour intensities. Future studies should aim to use device-based measures all parts of the movement behaviour composition in conjunction with self-reported time-use logs to reduce the potential for misclassification. Researchers should also consider collecting repeated measures of the movement behaviour composition to better capture habitual movement patterns over time and further elucidate temporal relationships.

## Conclusions

CoDA can help provide insight into the co-dependent relationships between movement behaviours and survival. In this study, the relative amount of time spent in MVPA and SED was significantly associated with a reduction and increase in mortality risk, respectively. Our estimates suggest replacing time spent in sleep, SED or LIPA with MVPA or replacing time spent in SED with any other movement behaviour would be associated with a lower mortality risk. Our results also suggest that insufficient sleep duration should be addressed by reallocating time into sleep from SED but not from LIPA or MVPA.

## Supplementary Information

**Additional file 1: Supplemental tables.**

## Data Availability

The NHANES datasets analyzed during the current study are freely and publicly available from the NHANES dedicated webpages hosted by the Center for Disease Control and Prevention. The main webpage https://www.cdc.gov/nchs/nhanes/index.htm provides access to the datasets, data documentation and information.

## Abbreviations

BMI: Body mass index
CoDA: Compositional data analysis
HR: Hazard ratio
*ilr*: Isometric log ratio
LIPA: Light intensity physical activity
MVPA: Moderate-to-vigorous intensity physical activity
NHANES: National Health and Nutrition Examination Survey
SED: Sedentary behaviour
