# Supplementary material for: A compositional analysis of time spent in sleep, sedentary behaviour and physical activity with all-cause mortality risk
Source: Int J Behav Nutr Phys Act. 2021 Feb 6;18:25. doi: 10.1186/s12966-021-01092-0 (PMC7866642; doi:10.1186/s12966-021-01092-0)
Supplement: Supplementary file 1 — Additional file 1: Supplemental tables. [file 12966_2021_1092_MOESM1_ESM.docx]

Supplemental Table 1. Estimated hazard ratios for displacing time between movement behaviours

| **Predicted hazard ratios** | | | | | | | | | | | | |
| --- | --- | --- | --- | --- | --- | --- | --- | --- | --- | --- | --- | --- |
|  |  |  |  |  |  |  |  |  |  |  |  |  |
| Remove time from: | Add time to MVPA (minutes) | | | | |  | Add time to: | Remove time from MVPA (minutes) | | | | |
|  | 15 | 30 | 45 | 60 | 75 |  |  | 15 | 30 | 60 | 90 | 120 |
| SED | 0.84 (0.78, 0.90) | 0.75 (0.67, 0.84) | 0.69 (0.59, 0.80) | 0.64 (0.53, 0.78) | 0.61 (0.49, 0.75) |  | SED | 1.80 (1.46, 2.22) |  |  |  |  |
| LIPA | 0.86 (0.82, 0.90) | 0.78 (0.73, 0.84) | 0.74 (0.67, 0.80) | 0.70 (0.64, 0.78) | 0.68 (0.61, 0.75) |  | LIPA | 1.76 (1.46, 2.13) |  |  |  |  |
| Sleep | 0.85 (0.81, 0.89) | 0.77 (0.71, 0.83) | 0.71 (0.65, 0.78) | 0.67 (0.61, 0.75) | 0.64 (0.57, 0.72) |  | Sleep | 1.78 (1.47, 2.15) |  |  |  |  |
|  |  |  |  |  |  |  |  |  |  |  |  |  |
| Remove time from: | Add time to LIPA (minutes) | | | | |  | Add time to: | Remove time from LIPA (minutes) | | | | |
|  | 15 | 30 | 60 | 90 | 120 |  |  | 15 | 30 | 60 | 90 | 120 |
| SED | 0.98 (0.96, 1.00) | 0.96 (0.92, 1.00) | 0.92 (0.84, 1.00) | 0.88 (0.77, 1.00) | 0.84 (0.71, 1.00) |  | SED | 1.02 (1.00, 1.04) | 1.04 (1.00, 1.09) | 1.09 (1.00, 1.19) | 1.14 (1.00, 1.30) | 1.19 (1.00, 1.42) |
| Sleep | 0.99 (0.99, 0.99) | 0.98 (0.98, 0.98) | 0.96 (0.96, 0.97) | 0.95 (0.95, 0.95) | 0.93 (0.93, 0.93) |  | Sleep | 1.01 (1.01, 1.01) | 1.02 (1.02, 1.03) | 1.04 (1.03, 1.06) | 1.07 (1.05, 1.09) |  |
| MVPA | 1.76 (1.46, 2.13) |  |  |  |  |  | MVPA | 0.86 (0.82, 0.90) | 0.78 (0.73, 0.84) | 0.70 (0.64, 0.78) |  |  |
|  |  |  |  |  |  |  |  |  |  |  |  |  |
| Remove time from: | Add time to SED (minutes) | | | | |  | Add time to: | Remove time from SED (minutes) | | | | |
|  | 15 | 30 | 60 | 90 | 120 |  |  | 15 | 30 | 60 | 90 | 120 |
| MVPA | 1.80 (1.46, 2.22) |  |  |  |  |  | MVPA | 0.84 (0.78, 0.90) | 0.75 (0.67, 0.84) | 0.64 (0.53, 0.78) |  |  |
| LIPA | 1.02 (1.00, 1.04) | 1.04 (1.00, 1.09) | 1.09 (1.00, 1.19) | 1.14 (1.00, 1.30) | 1.19 (1.00, 1.42) |  | LIPA | 0.98 (0.96, 1.00) | 0.96 (0.92, 1.00) | 0.92 (0.84, 1.00) | 0.88 (0.77, 1.00) | 0.84 (0.71, 1.00) |
| Sleep | 1.01 (0.99, 1.03) | 1.02 (0.98, 1.06) | 1.04 (0.97, 1.13) | 1.06 (0.95, 1.19) | 1.08 (0.93, 1.27) |  | Sleep | 0.99 (0.97, 1.01) | 0.98 (0.94, 1.02) | 0.95 (0.88, 1.03) | 0.93 (0.83, 1.04) |  |
|  |  |  |  |  |  |  |  |  |  |  |  |  |
| Remove time from: | Add time to sleep (minutes) | | | | |  | Add time to: | Remove time from sleep (minutes) | | | | |
|  | 15 | 30 | 60 | 90 | 120 |  |  | 15 | 30 | 60 | 90 | 120 |
| SED | 0.99 (0.97, 1.01) | 0.98 (0.94, 1.02) | 0.95 (0.88, 1.03) | 0.93 (0.83, 1.04) |  |  | SED | 1.01 (0.99, 1.03) | 1.02 (0.98, 1.06) | 1.04 (0.97, 1.13) | 1.06 (0.95, 1.19) | 1.08 (0.93, 1.27) |
| LIPA | 1.01 (1.01, 1.01) | 1.02 (1.02, 1.03) | 1.04 (1.03, 1.06) | 1.07 (1.05, 1.09) |  |  | LIPA | 0.99 (0.99, 0.99) | 0.98 (0.98, 0.98) | 0.96 (0.96, 0.97) | 0.95 (0.95, 0.95) | 0.93 (0.93, 0.93) |
| MVPA | 1.78 (1.47, 2.15) |  |  |  |  |  | MVPA | 0.85 (0.81, 0.89) | 0.77 (0.71, 0.83) | 0.67 (0.61, 0.75) |  |  |

LIPA, light intensity physical activity; MVPA, moderate-to-vigorous intensity physical activity; SED, sedentary behaviour

Note: Data presented as hazard ratio (95% confidence interval). All estimates have been adjusted for age, gender, education, family poverty-to-income ratio, BMI, smoking status, alcohol consumption, and diet quality. Hazard ratios reflect the estimated change in mortality risk associated with reallocating time increments between movement behaviours using the mean movement behaviour composition as the reference.

Supplemental Table 2. Estimated hazard ratios for displacing time between one and the remaining movement behaviours proportionally

| **Predicted hazard ratios** | | | | | | | | | | | | | |
| --- | --- | --- | --- | --- | --- | --- | --- | --- | --- | --- | --- | --- | --- |
|  |  |  |  |  |  |  |  |  |  |  |  |  |  |
| Remove time from | Minutes | | | | |  |  | Add time to | Minutes | | | | |
|  | 15 | 30 | 60 | 90 | 120 |  |  |  | 15 | 30 | 60 | 90 | 120 |
| MVPA | 1.78 (1.46, 2.17) |  |  |  |  |  |  | MVPA | 0.85 (0.80, 0.90) | 0.76 (0.70, 0.84) | 0.67 (0.58, 0.77) |  |  |
| LIPA | 1.01 (1.00, 1.03) | 1.03 (1.00, 1.05) | 1.06 (1.00, 1.11) | 1.09 (1.01, 1.18) | 1.12 (1.01, 1.25) |  |  | LIPA | 0.99 (0.98, 1.00) | 0.98 (0.95, 1.00) | 0.95 (0.91, 1.00) | 0.93 (0.87, 0.99) | 0.91 (0.83, 0.99) |
| SB | 0.98 (0.96, 1.00) | 0.96 (0.92, 1.00) | 0.92 (0.84, 1.00) | 0.88 (0.77, 1.01) | 0.84 (0.70, 1.01) |  |  | SB | 1.02 (1.00, 1.04) | 1.04 (1.00, 1.09) | 1.09 (1.00, 1.18) | 1.13 (1.00, 1.28) | 1.18 (0.99, 1.40) |
| Sleep | 1.00 (0.99, 1.01) | 1.00 (0.98, 1.02) | 1.00 (0.96, 1.04) | 0.99 (0.93, 1.06) | 0.99 (0.91. 1.08) |  |  | Sleep | 1.00 (0.99, 1.01) | 1.00 (0.98, 1.02) | 1.00 (0.97, 1.04) | 1.01 (0.95, 1.06) |  |

LIPA, light intensity physical activity; MVPA, moderate-to-vigorous intensity physical activity; SED, sedentary behaviour

Note: Data presented as hazard ratio (95% confidence interval). All estimates have been adjusted for age, gender, education, family poverty-to-income ratio, BMI, smoking status, alcohol consumption and diet quality. Hazard ratios reflect the estimated change in mortality risk associated with reallocating time increments from one movement behaviour to the remaining movement behaviours proportionally (or vice versa) using the mean movement behaviour composition as the reference.
